# Coexistence of *tmexCD-toprJ*, *bla*_NDM-1_, and *bla*_PME-1_ in multi-drug-resistant *Pseudomonas juntendi* isolates recovered from stool samples

**DOI:** 10.1128/spectrum.01136-24

**Published:** 2025-02-25

**Authors:** Wugao Liu, Yi Liu, Jing Jin, Ningjun Wu, Weiping Wu, Chunsheng Qu

**Affiliations:** 1 Clinical Laboratory Lishui People’s Hospital, The Sixth Affiliated Hospital of Wenzhou Medical University, Lishui, China; 2 Collaborative Innovation Center for Diagnosis and Treatment of Infectious Diseases, State Key Laboratory for Diagnosis and Treatment of Infectious Diseases, the First Affiliated Hospital, Zhejiang University School of Medicine, Hangzhou, China; Ross University School of Veterinary Medicine, Basseterre, Saint Kitts and Nevis

**Keywords:** *Pseudomonas juntendi*, integrative and conjugative element, *tmexCD-toprJ*, *bla*_NDM-1_, *bla*_PME-1_

## Abstract

*Pseudomonas juntendi* has received limited research attention, yet strains carrying multi-drug resistance genes pose a threat to global public health. We aimed to characterize the genome of two fecal-derived strains of *Pseudomonas juntendi*, both harboring *tmexCD-toprJ*, *bla*_NDM-1_, and *bla*_PME-1_ on the chromosome, recovered from two patients. Average nucleotide identity (ANI) analysis showed that L4008hy and L4046hy were remarkably similar. They showed high levels of resistance to aztreonam, imipenem, ciprofloxacin, amikacin, piperacillin–tazobactam, ceftazidime–avibactam, and polymyxin B in antimicrobial susceptibility testing using agar dilution method and broth microdilution methods. Additionally, an integrative and conjugative element (ICE) similar to ICE*6660* was detected on the chromosome, which contains all resistance genes and has a relatively complete transfer module, and potential transfer mechanisms were identified. Phylogenetic analysis of *P. juntendi* reveals the genomic diversity of the species and sheds light on environmental–human transmission.

**IMPORTANCE:**

Up to now, research on *Pseudomonas juntendi* is still very limited. Our findings suggest that *P. juntendi* commonly carries diversity resistance genes on chromosomes and is stably inherited, highlighting the need for further studies on the antimicrobial properties of this bacterium. The coexistence of *tmexCD-toprJ*, *bla*_NDM-1_, and *bla*_PME-1_ on the chromosome in *P. juntendi* was reported for the first time. The identified integrative and conjugative element (ICE) contains all the identified resistance genes and serves as a vector for resistance gene transfer between bacteria. *P. juntendi*, which harbors multi-drug resistance genes, particularly those encoding carbapenemases, acts as a reservoir of resistance genes. Its spread in clinical settings poses additional challenges to treatment.

## OBSERVATION

*Pseudomonas* spp. are Gram-negative bacteria with a remarkable metabolic diversity and extensive environmental adaptability, enabling them to colonize a wide range of hosts (1), which are commonly isolated from environmental sources, such as plants and soil (2). Several species within the *Pseudomonas* genus have been identified as opportunistic pathogens, with *Pseudomonas aeruginosa* being the most frequently reported. Another member, *Pseudomonas putida*, belonging to a phylogenetic subgroup of *Pseudomonas* spp., has also been implicated in nosocomial infections and outbreaks, posing a significant threat to global public health (3–5). Treatment challenges arise particularly when *P. putida* acquires resistance genes through horizontal gene transfer, including carbapenem resistance. Notably, members of the *P. putida* group harbor a considerable arsenal of resistance genes, acting as reservoirs for these genes. These genes can be disseminated between bacteria via plasmids, mobile genetic elements, and integrative conjugative elements (ICEs), further complicating infection control and treatment strategies. Within the *P. putida* group, newly designated species, such as *Pseudomonas asiatica* and *Pseudomonas juntendi*, have recently been linked to human infections (6). *P. juntendi* was first isolated in 2019 from sputum samples of patients in Japan and Myanmar (6). However, studies on the antimicrobial resistance of *P. juntendi* remain limited to date.

**Fig 1:**
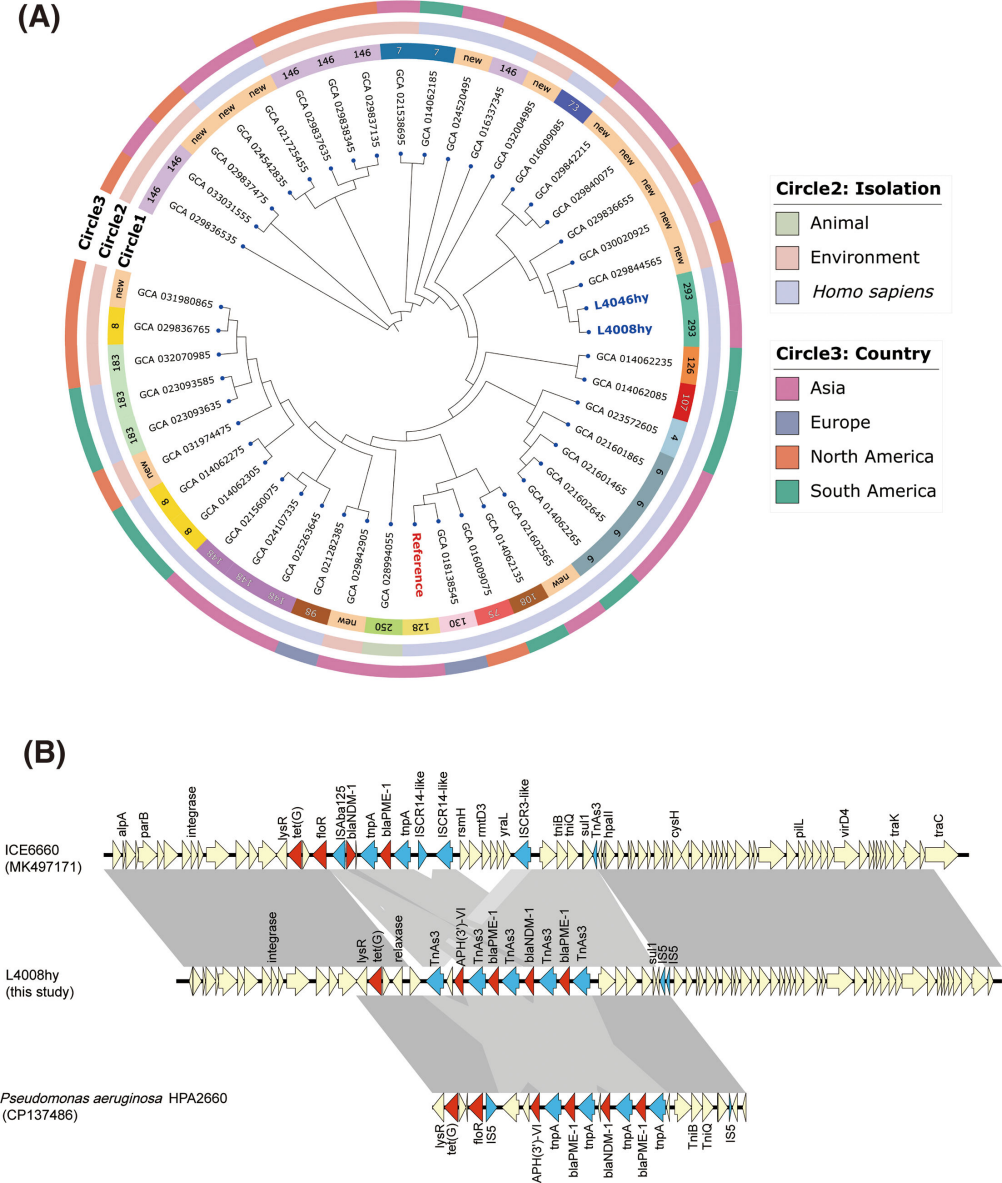
Genetic comparison and phylogenetic tree analysis of strains L4008hy and L4046hy. (**A**) Forty-five *P*. *juntendi* strains deposited in the NCBI database and two strains in our collection were included in this phylogenetic tree. Circle 1 depicts the ST types of these strains. Circle 2 denotes the isolations of strains. Circle 3 denotes the country of strains. (**B**) Comparison of genes surrounding *bla*_NDM-1_ and *bla*_PME-1_ on L4008hy, ICE6660 (MK497171) and *Pseudomonas aeruginosa* HPA2660 (CP137486) are shown as arrows and indicated according to their putative functions. Red indicates antimicrobial resistance genes, blue indicates mobile elements, yellow represents other functional genes. Regions with a high degree of homology are indicated by grey shading.

In this study, we report the genomes of two multidrug-resistant *P. juntendi* isolates, L4008hy and L4046hy, recovered from the stool samples of two patients. In July 2021, isolate L4008hy was obtained from the fecal sample of a 46-year-old male patient at the First Affiliated Hospital of Zhejiang University. Subsequently, isolate L4046hy was recovered from the fecal sample of a 67-year-old female patient. Initially, both isolates were identified as *Pseudomonas* spp. using Matrix-Assisted Laser Desorption/Ionization-Time-of-Flight Mass Spectrometry (MALDI-TOF MS). However, sequencing and average nucleotide identity (ANI) analysis revealed that L4008hy and L4046hy share more than 97% similarity with the *P. juntendi* reference strain BML3 (accession number: GCA_009932375.1), confirming their classification as *P. juntendi*. Despite being isolated from different patients, the genomic features of the two strains were remarkably similar. BLAST analysis between L4008hy and L4046hy demonstrated 98% coverage and 99.98% sequence identity. In addition, 122 SNPs were identified between the two strains, but they belong to the same phylogenetic branch (Fig. 1A), suggesting that the two strains are not clonal but may have evolved from a common ancestor. Therefore, further genomic analysis was conducted using L4008hy as the representative strain.

L4008hy was classified as ST293, a sequence type that has not been previously reported in *P. juntendi*. The resistance genes of L4008hy were located on a 5,608,759 bp chromosome. This strain harbors a diverse array of resistance genes, including *bla*_NDM-1_, *bla*_PME-1_, *tmexCD-toprJ*, *aph(3′)-VIa*, *sul1*, *tet(G*), and *floR*. Antimicrobial susceptibility testing (AST) revealed that L4008hy exhibited resistance to multiple antibiotics, including aztreonam, imipenem, meropenem, ceftazidime, levofloxacin, ciprofloxacin, amikacin, piperacillin–tazobactam, cefepime, ceftazidime–avibactam, polymyxin B (Table 1). It has been reported that mutations in *gyrA*, *gyrB*, and *parC* can confer resistance to ciprofloxacin in *P. aeruginosa* (7, 8). Interestingly, missense mutations in *gyrA* (G898D and T83I), *gyrB* (N640S), and *parC* (G535C and V336I) were also identified in the ciprofloxacin-resistant strain L4008hy.

**TABLE 1:** MICs of *P. juntendi* L4008hy, L4046hy, and control strain ATCC 25922

	MIC (mg/L)		
	L4008hy	L4046hy	ATCC 25922
Aztreonam	>128(R) ^*a*^	128(R)	0.25(S)
Imipenem	>32(R)	>32(R)	0.5(S)
Meropenem	>32(R)	>32(R)	0.03(S)
Ceftazidime	>128(R)	>128(R)	0.5(S)
Levofloxacin	>64(R)	32(R)	0.03(S)
Ciprofloxacin	>64(R)	16(R)	0.015(S)
Amikacin	64(R)	64(R)	4(S)
Piperacillin–tazobactam	>128(R)	>128(R)	4(S)
Cefepime	>128(R)	>128(R)	0.06(S)
Ceftazidime–avibactam	>64(R)	>64(R)	0.5(S)
Polymyxin B	>8(R)	>8(R)	0.5(S)
Ceftriaxone	>128(NA)	>128(NA)	0.06(S)
Cefotaxime	>128(NA)	>128(NA)	0.125(S)
Gentamicin	8(NA)	8(NA)	1(S)
Chloramphenicol	>128(NA)	>128(NA)	8(S)
Amoxicillin–clavulanic acid	>128(NA)	>128(NA)	4(S)
Tigecycline	4(NA)	4(NA)	<0.06(S)
Eravacycline	2(NA)	1(NA)	0.125(S)
Omadacycline	32(NA)	16(NA)	0.25(S)

^*a*^ SIR, susceptible (S), intermediate (I), resistant (R), not applicable (NA).

IncX3 and InA/C plasmids are known to facilitate the transmission of the *bla*_NDM_ gene; however, in L4008hy and L4046hy, *bla*_NDM-1_ was found integrated into the chromosome (9, 10). Moreover, Pfeifer et al. (11) reported that *bla*_NDM-1_ in *Acinetobacter baumannii* could integrate into the chromosome via IS*26*, enhancing the stable inheritance of the gene. This demonstrates that *bla*_NDM-1_ is not only transmissible between bacteria through plasmids but can also integrate into the bacterial chromosome by mobile elements, thereby ensuring more stable antibiotic resistance. In the isolates L4008hy and L4046hy, *bla*_NDM-1_ is surrounded by two *bla*_PME-1_ copies and connected via Tn*As3*, forming a composite structure: Tn*As3-bla*_PME-1_–Tn*As3-bla*_NDM-1_–Tn*As3-bla*_PME-1_–Tn*As3*. Additionally, the structure is flanked by mobile elements, such as IS*5*, both upstream and downstream, which facilitate the transfer of the entire composite structure. A similar complex arrangement was also observed in *P. aeruginosa* strain HPA2660 (accession number: CP137486), where the surrounding genomic environment of the resistance genes is identical to that of L4008hy (Fig. 1B). Bao et al. (12) were the first to report the coexistence of *bla*_NDM-1_ and two *bla*_PME-1_ copies in *P. juntendi*, linked by IS*91*. This suggests that horizontal gene transfer of resistance genes occurs between *P. aeruginosa* and *P. juntendi*. Although the exact mechanism of spread remains unclear, mobile elements appear to play a critical role in this process.

In recent years, with the rise of carbapenem and polymyxin resistance, tigecycline has been considered to be the last line of defense against multidrug-resistant infections in humans. The *tmexCD-toprJ,* carried on the L4008hy chromosome, which confers tigecycline resistance, has received considerable attention. Besides conferring resistance to tigecycline, *tmexCD-toprJ* also showed resistance to several antibiotics, such as ciprofloxacin, chloramphenicol, eravacycline, and cefotaxime, consistent with the AST result of L4008hy. Plasmid-mediated horizontal transfer of *tmexCD-toprJ* was more common, with its occurrence on the chromosome (e.g., ICEs) (13, 14). It is worth noting that the *tmexCD-toprJ* can coexist with carbapenem-resistant genes and *mcr* genes, which undoubtedly exacerbates the current state of bacterial resistance (15–17).

Apart from conjugative plasmids, the ICEs within chromosomes also mediate conjugative transfer (18). ICEs undergo horizontal gene transfer (HGT) through transfer function modules, such as oriT, relaxase, T4CP, and T4SS. The chromosome of L4008hy contains several T4SS-type ICEs, among which a 128.55 kb T4SS-type ICE (designated ICE_L4008) has attracted our attention. ICE_L4008 has a GC content of 63.47% and contains all resistance genes on the chromosome. ICE_L4008 has complete integrases and relaxases, along with transfer system modules T4CP and T4SS. The attachment sites *attL* (3,982,851...3,982,865) and *attR* (4,111,388...4,111,402) flanked the resistant genes, although the origin of transfer *oriT* and the cleavage enzyme *xis* are absent. This suggests that the transfer of resistant genes present on chromosome L4008hy may occur via the ICE structure. Furthermore, ICE_L4008 shows high homology (with 52% coverage and 100% identity) to ICE6660 (GenBank accession: MK497171) found in *P. aeruginosa,* as observed by mlutigeneblast comparison (Fig. 1B).

The phylogenetic relationships of *P. juntendi* were investigated using a phylogenetic tree (Fig. 1A; Table S1). The phylogenetic tree was constructed using the maximum-likelihood method after SNP analysis of 45 genomes available on NCBI (as of March 2024), using the reference strain *P. juntendi* BML3. With the exception of L4008hy and L4046hy, the other strains within the same branch were predominantly isolated from environmental samples, indicating a trend towards environmental–human transmission of *P. juntendi* without geographical concordance. Regarding the region of isolation, 42.6% were isolated from Asia (20/47). The global genome of *P. juntendi* showed diversity, comprising 47 strains belonging to 17 ST types, with ST146 (6/47) and ST6 (4/47) being the most common, and 14 strains representing novel types. Interestingly, in addition to L4008hy and L4046hy, *bla*_NDM-1_ was detected in three strains, all of which coexisted with *bla*_PME-1_. These strains were isolated from China and Pakistan, suggesting that the co-occurrence of *bla*_NDM-1_ and *bla*_PME-1_ has established a pattern of resistance in *P. juntendi*.

In this study, we report the genomes of two isolates of multidrug-resistant *P. juntendi* from two patient’s stool samples and identify several resistance genes, including *bla*_NDM-1_, *bla*_PME-1_, *tmexCD-toprJ*, *aph(3′)-VIa*, *sul1*, *tet(G*), and *floR* in *P. juntendi* strains. To our knowledge, this is the first time that the coexistence of *bla*_NDM-1_, *bla*_PME-1_, and *tmexCD-toprJ* has been identified in *P. juntendi*. We also investigated the biological evolution of the strains by constructing phylogenetic trees. Intriguingly, all identified resistance genes are located within an ICE6660-like ICE on the chromosome. Our findings suggest that *P. juntendi* may serve as a reservoir of resistance genes, which can be transferred via ICE, facilitating environment-to-human transmission.

## Data Availability

The complete sequence of *P. juntendi* L4008hy and L4046hy has been submitted to GenBank under accession no. CP146690-CP146692.

## SUPPLEMENTAL MATERIAL

Supplemental material spectrum.01136-24-s0001.docx Supplemental materials and methods.

Table S1 spectrum.01136-24-s0002.docx Genomic information of 47 *P. juntendi* strains used to construct the phylogenetic tree.

ASM does not own the copyrights to Supplemental Material that may be linked to, or accessed through, an article. The authors have granted ASM a non-exclusive, world-wide license to publish the Supplemental Material files. Please contact the corresponding author directly for reuse.
