# Supplementary material for: Coexistence of tmexCD-toprJ, blaNDM-1, and blaPME-1 in multi-drug-resistant Pseudomonas juntendi isolates recovered from stool samples
Source: Microbiol Spectr. 2025 Feb 25;13(4):e01136-24. doi: 10.1128/spectrum.01136-24 (PMC11960068; doi:10.1128/spectrum.01136-24)
Supplement: Supplemental material — s and methods. [file spectrum.01136-24-s0001.docx]

**Materials and methods**

**Strains isolation and antimicrobial susceptibility testing**

L4008hy and L4046hy was isolated from the stool samples in First Affiliated Hospital of Zhejiang University, China. MALDI-TOF/MS initially identified the strains as *Pseudomonas spp.*. Subsequently, carbapenem resistance was detected using PCR and whole genome sequencing (WGS) was used to finally identify L4008hy and L4046hy as *Pseudomonas juntendi*.

The agar dilution method was used to determine the minimum inhibitory concentration (MIC) of the strain against aztreonam, imipenem, meropenem, ceftazidime, levofloxacin, ciprofloxacin, amikacin, piperacillin-tazobactam, cefepime, ceftazidime-avibactam, ceftriaxone, cefotaxime, gentamicin, chloramphenicol, amoxicillin-clavulanic acid. Additionally, the microbroth dilution method was utilized to determine the MIC values of the strain against omadacycline, eravacycline, tigecycline, polymyxin B, eravacycline, and omadacycline. AST results were interpreted based on the breakpoints of the CLSI (https://clsi.org), EUCAST (https://www.eucast.org/), and FDA criteria (https://www.fda.gov/drugs/development-resources/antibacterial-susceptibility-test-interpretive-criteria).

**WGS and data analysis**

DNA extraction of L4008hy and L4046hy was performed using the Genomic DNA Isolation Kit (QIAGEN, Hilden, Germany). The genomes of L4008hy and L4046hy were obtained from short reads and long reads generated by Illumina NovaSeq 6000 (Illumina, San Diego, CA, United States) and Oxford Nanopore platforms (Oxford Nanopore Technologies, Oxford, United Kingdom). Genome assembly was performed by Unicycler (1). Gene function prediction and annotation were performed by Prokka (2). Sequence type (ST) typing was determined by pubMLST (https://pubmlst.org/). The databases Resfinder (http://genepi.food.dtu.dk/resfinder) and VFDB (3) identified resistance genes and virulence genes in the genome. Insertion elements and transposons were analyzed by ISfinder (4). ANI analysis was performed by fastANI (<https://github.com/ParBLiSS/FastANI>) with *P. juntendi* strain BML3 (GCA_009932375) as the reference genome. SNP identification and phylogenetic tree were done by CSI Phylogeny (https://cge.food.dtu.dk/services/CSIPhylogeny/) and Snippy (https://github.com/tseemann/snippy), and the phylogenetic tree was modified by iTOL (https://itol.embl.de/). ICEberg 2.0 (5) was performed for the identification of ICEs and transfer function modules on the chromosome. Comparison of the environment around resistance genes was imaged by Easyfig (6).

1. Wick RR, Judd LM, Gorrie CL, Holt KE. 2017. Unicycler: Resolving bacterial genome assemblies from short and long sequencing reads. PLoS Comput Biol 13:e1005595.

2. Seemann T. 2014. Prokka: rapid prokaryotic genome annotation. Bioinformatics 30:2068-9.

3. Chen L, Yang J, Yu J, Yao Z, Sun L, Shen Y, Jin Q. 2005. VFDB: a reference database for bacterial virulence factors. Nucleic Acids Res 33:D325-8.

4. Siguier P, Perochon J, Lestrade L, Mahillon J, Chandler M. 2006. ISfinder: the reference centre for bacterial insertion sequences. Nucleic Acids Res 34:D32-6.

5. Liu M, Li X, Xie Y, Bi D, Sun J, Li J, Tai C, Deng Z, Ou HY. 2019. ICEberg 2.0: an updated database of bacterial integrative and conjugative elements. Nucleic Acids Res 47:D660-D665.

6. Sullivan MJ, Petty NK, Beatson SA. 2011. Easyfig: a genome comparison visualizer. Bioinformatics 27:1009-10.
