# Supplementary material for: Coexistence of tmexCD-toprJ, blaNDM-1, and blaPME-1 in multi-drug-resistant Pseudomonas juntendi isolates recovered from stool samples
Source: Microbiol Spectr. 2025 Feb 25;13(4):e01136-24. doi: 10.1128/spectrum.01136-24 (PMC11960068; doi:10.1128/spectrum.01136-24)
Supplement: Table S1 — Genomic information of 47 P. juntendi strains used to construct the phylogenetic tree. [file spectrum.01136-24-s0002.docx]

Table S1. Genomic information of 47 *P. juntendi* strains used to construct the phylogenetic tree

| FILE | ISOLATION | COUNTRY | REGION | ST | IMP | OXA | VIM | BKC | CRAB | PME | NDM | KPC |
| --- | --- | --- | --- | --- | --- | --- | --- | --- | --- | --- | --- | --- |
| GCA_014062085 | Homo sapiens | South America | Brazil | 107 | blaIMP-16 |  | blaVIM-2 |  |  |  |  |  |
| GCA_014062135 | Homo sapiens | South America | Brazil | 108 | blaIMP-1 |  |  |  |  |  |  |  |
| GCA_014062185 | Homo sapiens | South America | Brazil | 7 | blaIMP-16 |  | blaVIM-2 |  |  |  |  |  |
| GCA_014062235 | Homo sapiens | South America | Brazil | 126 | blaIMP-16 | blaOXA-129 | blaVIM-2 |  |  |  |  |  |
| GCA_014062265 | Homo sapiens | South America | Brazil | 6 |  |  |  | blaBKC-1 |  |  |  |  |
| GCA_014062275 | Homo sapiens | South America | Brazil | 8 |  | blaOXA-129 | blaVIM-2 |  |  |  |  |  |
| GCA_014062305 | Homo sapiens | South America | Brazil | 8 |  | blaOXA-129 | blaVIM-2 |  |  |  |  |  |
| GCA_016009075 | Homo sapiens | North America | USA | 75 |  |  |  |  |  |  |  |  |
| GCA_016009085 | Homo sapiens | North America | USA | 73 |  |  |  |  |  |  |  |  |
| GCA_016337345 | Homo sapiens | North America | USA | 146 |  |  |  |  |  |  |  |  |
| GCA_018138545 | Homo sapiens | Europe | Russia | 130 |  |  |  |  |  |  |  |  |
| GCA_021282385 | Homo sapiens | Europe | Poland | 98 |  |  | blaVIM-2 |  | blaCARB-2 |  |  |  |
| GCA_021538695 | Environment | Asia | India | 7 |  |  | blaVIM-2 |  |  |  |  |  |
| GCA_021560075 | Homo sapiens | Asia | China：Sanmen | 148 | blaIMP-15 |  |  |  | blaCARB-2 | blaPME-1 | blaNDM-1 |  |
| GCA_021601465 | Homo sapiens | Asia | Japan | 6 |  |  |  |  |  |  |  |  |
| GCA_021601865 | Homo sapiens | Asia | Japan | 6 |  |  |  |  |  |  |  |  |
| GCA_021602565 | Homo sapiens | Asia | Japan | new |  |  |  |  |  |  |  |  |
| GCA_021602645 | Homo sapiens | Asia | Japan | 6 |  |  |  |  |  |  |  |  |
| GCA_021725455 | Homo sapiens | Asia | China | new | blaIMP-1 |  |  |  |  |  |  |  |
| GCA_023093585 | Homo sapiens | South America | Brazil | 183 |  | blaOXA-10 |  |  |  |  |  | blaKPC-2 |
| GCA_023093635 | Homo sapiens | South America | Brazil | 183 |  | blaOXA-10 |  |  |  |  |  | blaKPC-2 |
| GCA_023572605 | Homo sapiens | South America | Brazil | 4 |  |  | blaVIM-2 |  |  |  |  |  |
| GCA_024107335 | Homo sapiens | Asia | China:Sanmen | 148 | blaIMP-15 |  |  |  | blaCARB-2 | blaPME-1 | blaNDM-1 |  |
| GCA_024520495 | Homo sapiens | Asia | China:Zhejiang | new | blaIMP-1 |  |  |  |  |  |  |  |
| GCA_024542835 | Homo sapiens | Asia | China:Changchun | new | blaIMP-1 |  |  |  |  |  |  |  |
| GCA_025263645 | Homo sapiens | Asia | China:Hunan | 148 |  |  |  |  |  |  |  |  |
| GCA_028994055 | Animal | Asia | China: Guangdong | 250 |  | blaOXA-10 |  |  |  |  |  |  |
| GCA_029836535 | Environment | North America | USA | 146 |  |  |  |  |  |  |  |  |
| GCA_029836655 | Environment | North America | USA | new |  |  |  |  |  |  |  |  |
| GCA_029836765 | Environment | North America | USA | 8 |  |  |  |  |  |  |  |  |
| GCA_029837135 | Environment | North America | USA | 146 |  |  |  |  |  |  |  |  |
| GCA_029837475 | Environment | North America | USA | new |  |  |  |  |  |  |  |  |
| GCA_029837635 | Environment | North America | USA | 146 |  |  |  |  |  |  |  |  |
| GCA_029838345 | Environment | North America | USA | 146 |  |  |  |  |  |  |  |  |
| GCA_029840075 | Environment | Asia | Pakistan | new |  | blaOXA-2 | blaVIM-2 |  |  |  |  |  |
| GCA_029842215 | Environment | Asia | Pakistan | new |  | blaOXA-2 | blaVIM-2 |  |  |  |  |  |
| GCA_029842905 | Environment | Asia | Pakistan | new |  |  |  |  |  |  |  |  |
| GCA_029844565 | Environment | North America | USA | new |  |  |  |  |  |  |  |  |
| GCA_030020925 | Environment | Asia | Malaysia | new |  |  |  |  |  |  |  |  |
| GCA_031974475 | Environment | North America | USA | new |  |  |  |  |  |  |  |  |
| GCA_031980865 | Environment | North America | USA | new |  |  |  |  |  |  |  |  |
| GCA_032004985 | Environment | North America | USA | new |  |  |  |  |  |  |  |  |
| GCA_032070985 | Environment | North America | USA | 183 |  |  |  |  |  |  |  |  |
| GCA_033031555 | Environment | Asia | Pakistan | 146 |  | blaOXA-2 | blaVIM-2 |  |  | blaPME-1 | blaNDM-1 |  |
| L4008hy | Homo sapiens | Asia | China:Zhejiang | 293 |  |  |  |  |  | blaPME-1 | blaNDM-1 |  |
| L4046hy | Homo sapiens | Asia | China:Zhejiang | 293 |  |  |  |  |  | blaPME-1 | blaNDM-1 |  |
| reference | Homo sapiens | Asia | Japan | 128 |  |  |  |  |  |  |  |  |
